# Pressure Overload-Induced Cardiac Hypertrophy Response Requires Janus Kinase 2-Histone Deacetylase 2 Signaling

**DOI:** 10.3390/ijms151120240

**Published:** 2014-11-05

**Authors:** Huang Ying, Mao-Chun Xu, Jing-Hua Tan, Jing-Hua Shen, Hao Wang, Dai-Fu Zhang

**Affiliations:** 1 Department of Cardiology, Shanghai Pu Dong New Area People’s Hospital, Shanghai 200120, China; E-Mails: yingyinghuang126@126.com (H.Y.); anjinghuahua@yeah.net (J.-H.T.); jinghua1981126@126.com (J.-H.S.); 2 Department of Cardiology, Huashan Hospital of Fudan University, Shanghai 200040, China; E-Mail: xumaochunchina@163.com; 3 Fudan University Shanghai Medical College Centre of Medical Experiments, Shanghai 200040, China

**Keywords:** pressure overload, cardiac hypertrophy, HDAC2, Jak2, Ang-II

## Abstract

Pressure overload induces cardiac hypertrophy through activation of Janus kinase 2 (Jak2), however, the underlying mechanisms remain largely unknown. In the current study, we tested whether histone deacetylase 2 (HDAC2) was involved in the process. We found that angiotensin II (Ang-II)-induced re-expression of fetal genes (Atrial natriuretic peptide (ANP) and brain natriuretic peptide (BNP)) in cultured cardiomyocytes was prevented by the Jak2 inhibitor AG-490 and HDAC2 inhibitor Trichostatin-A (TSA), or by Jak2/HDAC2 siRNA knockdown. On the other hand, myocardial cells with Jak2 or HDAC2 over-expression were hyper-sensitive to Ang-II. *In vivo*, pressure overload by transverse aorta binding (AB) induced a significant cardiac hypertrophic response as well as re-expression of *ANP* and *BNP* in mice heart, which were markedly reduced by AG-490 and TSA. Significantly, AG-490, the Jak2 inhibitor, largely suppressed pressure overload-/Ang-II-induced HDAC2 nuclear exportation *in vivo* and *in vitro*. Meanwhile, TSA or HDAC2 siRNA knockdown reduced Ang-II-induced *ANP*/*BNP* expression in Jak2 over-expressed H9c2 cardiomyocytes. Together, these results suggest that HDAC2 might be a downstream effector of Jak2 to mediate cardiac hypertrophic response by pressure overload or Ang-II.

## 1. Introduction

Pressure overload-induced cardiac hypertrophy is mediated partly by activation of Janus kinase 2 (Jak2) [1,2]. Under biochemical and/or mechanic stresses, cardiac Jak2 is activated mainly through the increased circulating angiotensin II (Ang-II) [3,4]. On the other hand, treatment with the Jak2 inhibitor AG-490 prevented the hypertrophic response, indicating that Jak2 is important in the development of cardiac hypertrophy [5].

Recent studies show that transverse aorta binding (AB)-induced cardiac hypertrophy is associated with increased expression/activity of histone deacetylases (HDACs) [2]. HDACs are post-translational modifying enzymes that can de-acetylate histones as well as non-histone proteins [6]. The dominant negative mutant of cardiac HDAC2 inhibited AB-induced cardiac hypertrophy response, indicating a critical role HDAC2 in the process [7,8,9]. Further, studies demonstrated that Trichostatin-A (TSA), a HDAC2 inhibitor, attenuated the development of cardiac hypertrophy [10,11]. Thus, HDAC2 is considered as an important signaling molecule for cardiac hypertrophy [9]. However, the underlying mechanisms are not fully understood, and its relationship with Jak2 is also not clear. In the current study, we found that HDAC2 might be a downstream effector of Jak2 to mediate cardiac hypertrophic response.

## 2. Results

### 2.1. Janus Kinase 2 (Jak2) and Histone Deacetylase 2 (HDAC2) Are Important for Angiotensin II (Ang-II)-Induced Atrial Natriuretic Peptide (ANP)/Brain Natriuretic Peptide (BNP) Expressions in H9c2 Cardiomyocytes

**Figure 1:**
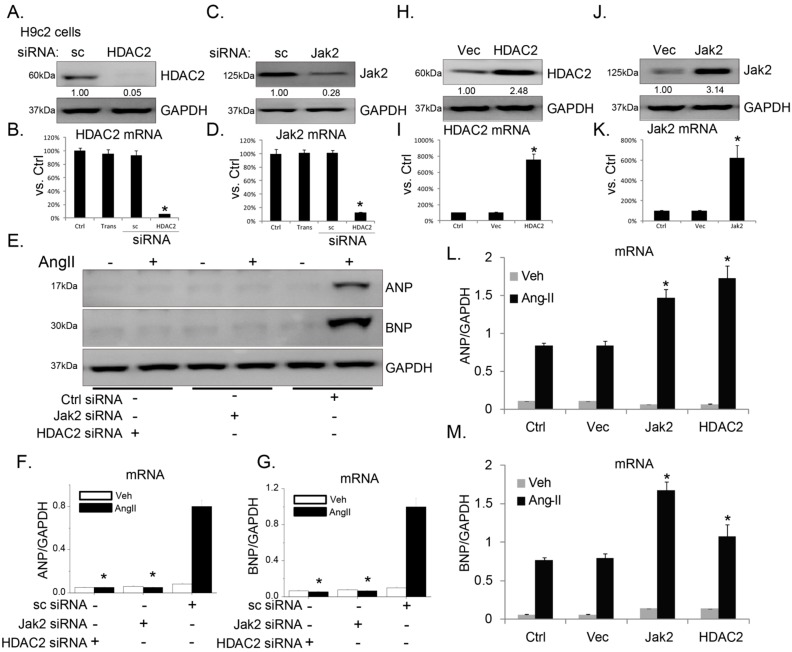
Janus kinase 2 (Jak2) and histone deacetylase 2 (HDAC2) are important for angiotensin II (Ang-II)-induced atrial natriuretic peptide (*ANP*)/brain natriuretic peptide (*BNP*) expression in H9c2 cardiomyocytes. Protein and mRNA expression of HDAC2 (**A**,**B**) or Jak2 (**C**,**D**) in H9c2 cells transfected with scramble siRNA (sc-siRNA) or indicated siRNA (100 nM each, 48 h); Protein (**E**) and mRNA (**F**,**G**) expression of *ANP*, *BNP* and glyceraldehyde-3-phosphate dehydrogenase (*GAPDH*) before and after Ang-II (1 μM, 24 h) treatment in H9c2 cells transfected with indicated siRNA; Protein (**H**) and mRNA expression of HDAC2 (**H**,**I**) and Jak2 (**J**,**K**) in stable H9c2 cells transfected with vector (“Vec”) or indicated cDNA plasmid (0.5 μg/mL each); Message RNA (mRNA) expression of *ANP* (**L**) and *BNP* (**M**) before and after Ang-II (1 μM, 24 h) treatment in stable H9c2 cells transfected with Jak2/HDAC2 plasmid or empty vector (“Vec”, 0.5 μg/mL each). For all the mRNA assays, *n* = 6. Experiments in this figure were repeated three times with similar results obtained. All data were expressed as means ± SEM, GAPDH was tested as an internal control. * *p* < 0.05 *vs.* “Ctrl” group (**B**,**D**,**I**,**K**); * *p* < 0.05 *vs.* Ang-II group of sc-siRNA transfected or non-transfected H9c2 cells (**F**,**G**,**L**,**M**). “Trans” stands for transfection reagent only. “Veh” stands for fetal bovine serum (PBS).

In order to test the role of Jak2 and HDAC2 in cardiac hypertrophy response, siRNA strategy was utilized to selectively knockdown Jak2 or HDAC2 in H9c2 cardiomyocytes. As shown in Figure 1A–D, protein and mRNA expression of HDAC2 or Jak2 was significantly down-regulated after targeted-siRNA transfection. Atrial natriuretic peptide (ANP) and brain natriuretic peptide (BNP) were synthesized and secreted mainly by atrial myocytes. In response to arterial hypertension, ventricular cardiomyocytes undergo phenotypic modifications that will result in the re-expression of fetal genes including *ANP* and *BNP* [12,13]. Thus, these two genes are detected as indicators of cardiac hypertrophy. Here we found that Ang-II induced protein and mRNA expression of *ANP* and *BNP* in H9c2 cells, which was significantly inhibited by Jak2 or HDAC2 siRNA knockdown (Figure 1E–G). On the other hand, Jak2 or HDAC2 over-expression (Figure 1H–K) enhanced Ang-II-induced *ANP*/*BNP* expression (Figure 1L,M). Based on these data, we suggest that Jak2 and HDAC2 are important for Ang-II-induced *ANP*/*BNP* expressions in H9c2 cells.

### 2.2. AG-490 or Trichostatin-A (TSA) Inhibits Ang-II-Induced Cardiac Hypertrophy Response in Primary Mouse Cardiomyocytes

**Figure 2:**
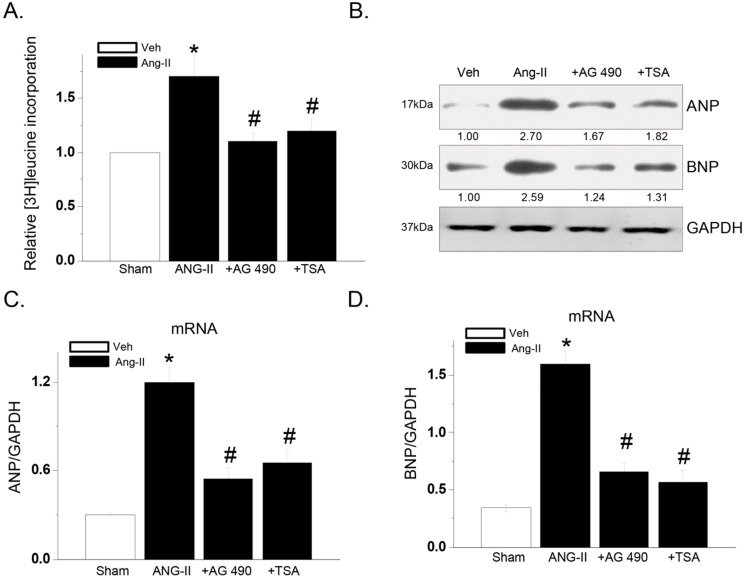
AG-490 or Trichostatin-A (TSA) inhibits Ang-II-induced cardiac hypertrophy response in primary mouse cardiomyocytes. Primary mouse cardiomyocytes were pretreated with AG-490 (10 μM) or TSA (100 ng/mL) for 1 h, followed by Ang-II (1 μM) stimulation for 24 h, [^3^H]-leucine incorporation was analyzed (**A**, *n* = 6); Protein and mRNA expressions of *ANP*, *BNP* and *GAPDH* were tested by Western blots (**B**) and real-time PCR (**C**,**D**, *n* = 6), respectively. Experiments in this figure were repeated three times with similar results obtained. All data were expressed as means ± SEM, GAPDH was tested as an internal control for PCR and Western blot assays. * *p* < 0.05 *vs.* “Sham (Saline)” group. **^#^** *p* < 0.05 *vs.* Ang-II only group. “Veh” stands for PBS.

To further confirm the role of HDAC2 and Jak2 in cardiac hypertrophy response, primary cultured mouse neonatal cardiomyocytes were utilized. Since these primary cells were difficult to transfect, we utilized pharmacological inhibitors to interfere with HDAC2 or Jak2 activity. As shown in Figure 2A,B, Ang-II-induced hypertrophic response in primary neonatal cardiomyocytes was detected by increased cellular protein synthesis (enhanced [^3^H]-leucine incorporation) (Figure 2A) and *ANP*/*BNP* expression (Figure 2B–D). AG-490, the Jak2 blocker [14] and Trichostatin-A (TSA), the HDAC2 inhibitor [15] dramatically inhibited Ang-II-induced protein synthesis (Figure 2A) and *ANP*/*BNP* expression (protein and mRNA) (Figure 2B–D) in primary neonatal cardiomyocytes. These findings once again suggest that both Jak2 and HDAC2 are important for hypertrophic responses induced by Ang-II in primary cardiomyocytes.

### 2.3. AG-490 or TSA Inhibits Pressure Overload-Induced Cardiac Hypertrophy in Vivo

**Figure 3:**
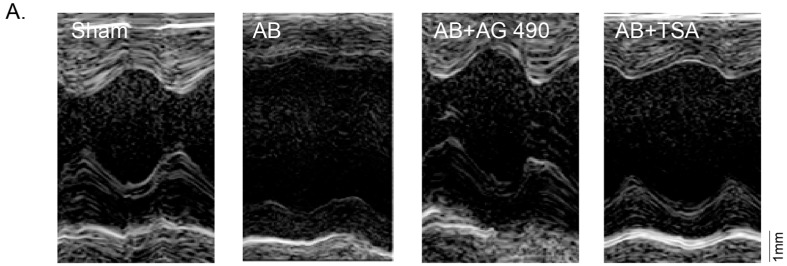

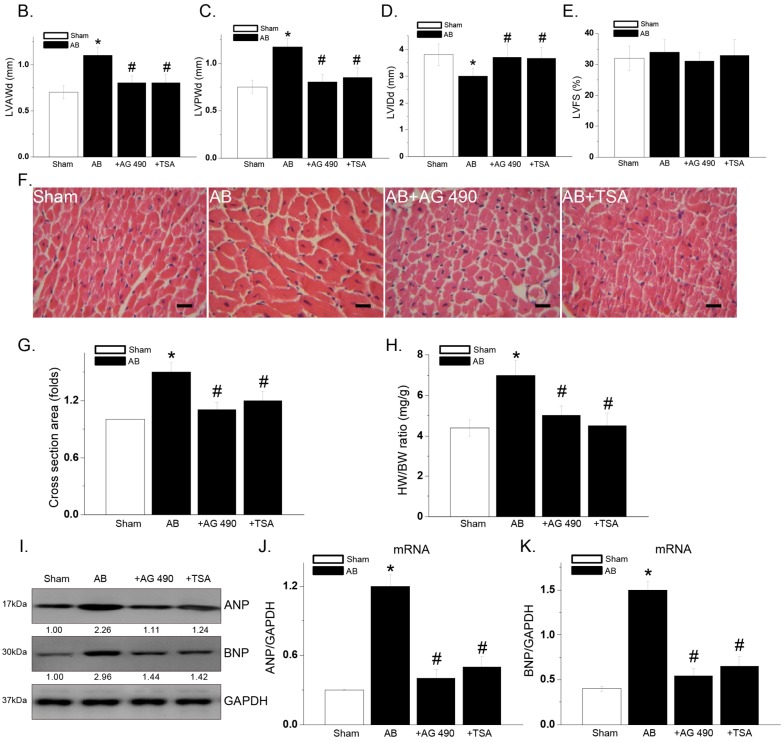
AG-490 or TSA inhibits pressure overload-induced cardiac hypertrophy *in vivo.* Mice (8–10 weeks, male, *n* = 5) were subjected to aorta binding (AB) or sham operations, with or without AG-490 (1 mg/kg body weight per day) or TSA (0.6 mg/kg body weight per day) for two weeks (see Material and Methods), after another two weeks recovery, representative M-mode tracings of echocardiography parameter analysis were shown (**A**–**E**); Representative hematoxylin and eosin (H&E) staining (scale bar: 20 µm) (**F**); cross-sectional area (CSA) (**G**) and heart weight to body weight (HW/BW) ratio (**H**) were also detected; Protein and mRNA expressions of *ANP*, *BNP* and *GAPDH* in above mouse left ventricular (LV) tissues were tested by Western blots (**I**) and real-time PCR (**J**,**K**, *n* = 6). Experiments in this figure were repeated three times with similar results obtained. All data were expressed as means ± SEM, GAPDH was served as an internal control for PCR and Western blot assays. * *p* < 0.05 *vs.* “Sham” operation group. **^#^** *p* < 0.05 *vs.* AB only group. LVAWd, LV anterior wall thickness at end-diastole; LVPWd, LV posterior wall thickness at end-diastole; LVIDd, LV internal dimension at end-diastole; LVEF, left ventricular eject fraction.

Above findings clearly demonstrated that Jak2 and HDAC2 were both involved in cardiac hypertrophic response induced by Ang-II. Next, we tested this hypothesis *in vivo*. Cardiac hypertrophy was induced through transverse aorta binding (AB) as described in Material and Methods. Two weeks after AB, all of the experimental mice survived, and were subjected to Transthoracic Echocardiogram (TTE) examination and hemodynamic examinations (Figure 3A–E). Compared with sham-operated mice, mice with AB for two weeks presented with similar left ventricular eject fraction (LVEF) (Figure 3E), but significant thicker left ventricular anterior and posterior wall at end-diastole (LVAWd, LVPWd) (Figure 3B,C), decreased left ventricular internal diameter at end-diastole (LVIDd) (Figure 3A,D). Meanwhile, heart weight/body weight (HW/BW) ratio (Figure 3H) and cardiomyocyte cross-sectional area (CSA) were also increased in experimental mice (Figure 3G). Morphological H&E images in Figure 3F confirmed the above observations. Such effects by AB were alleviated by treatment with Jak2 inhibitor AG-490 or HDAC2 inhibitor TSA (Figure 3A–H). Moreover, AG-490 and TSA inhibited mRNA and protein expression of *ANP* and *BNP* in AB mice heart left ventriculars (LVs) (Figure 3I–K). Together, these results suggest that HDAC2 and Jak2 function as critical intracellular signaling molecules in the stress-induced cardiac hypertrophic responses.

### 2.4. Jak2 Is Required for HDAC2 Nuclear Exportation-Induced by Pressure Overload and Ang-II

**Figure 4:**
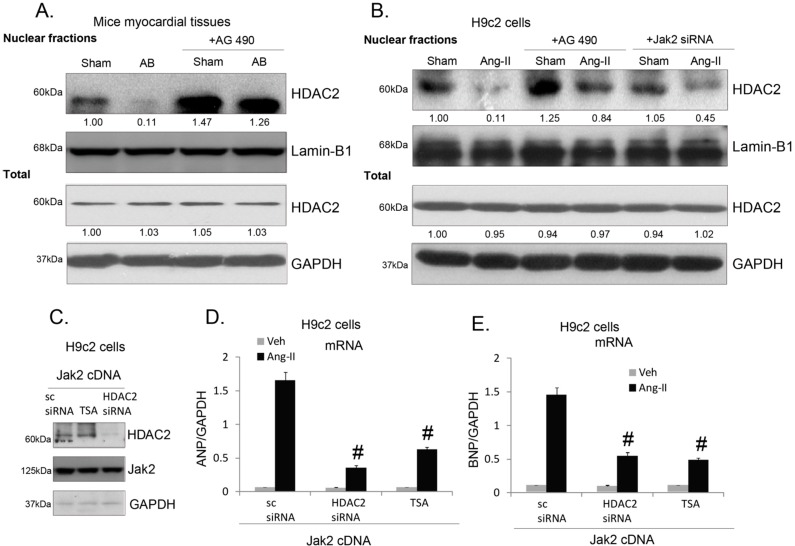
Jak2 is required for HDAC2 nuclear exportation-induced by pressure overload and Ang-II. Nuclear *HDAC2* and *Lamin-B1* expressions in myocardial tissue of mice subjected to AB or sham operations for two weeks, with or without AG-490 (1 mg/kg body weight per day) or TSA (0.6 mg/kg body weight per day), were tested by Western blots, total *HDAC2* and *GAPDH* expressions were also tested (**A**); H9c2 cells were pretreated with AG-490 (10 μM, 1 h) or Jak2 siRNA (100 nM, 48 h), followed by Ang-II (1 μM) stimulation for 12 h, nuclear *HDAC2* and *Lamin-B1* expressions were tested, total *HDAC2* and *GAPDH* expressions were also tested (**B**); Stable H9c2 cells over-expressing Jak2 were transfected with scramble siRNA (sc-siRNA) or HDAC2 siRNA (100 nM each, 48 h), cells were further treated with Ang-II (1 μM) in the presence or absence of TSA (100 ng/mL) for 24 h, mRNA expressions of *ANP*/*BNP* (*vs.* *GAPDH*) were tested (**D**,**E**, *n* = 6); protein expressions of *HDAC2*, *Jak2* and *GAPDH* were tested by Western blots (**C**); Experiments in this figure were repeated three times with similar results obtained. All data were expressed as means ± SEM. ^#^ *p* < 0.05 *vs.* Ang-II treatment with sc-siRNA. “Veh” stands for PBS.

The above evidence indicates that Jak2 and HDAC2 are important for pressure overload-induced cardiac hypertrophy response *in vitro* and *in vivo*. Next, we explored the relationship between the two. We observed that AB pressure overload induced HDAC2 nuclear exportation in the mice heart LV tissues, which was prevented by Jak2 inhibitor AG-490 (Figure 4A), suggesting that Jak2 might be required for HDAC2 nuclear exportation. Further, in H9c2 cells, HDAC2 nuclear exportation by Ang-II was inhibited by AG-490 or Jak2 siRNA knockdown (Figure 4B). Note that the total HDAC2 expression was not affected by JAK2 inhibition (Figure 4A,B). Ang-II-induced *ANP*/*BNP* expression in Jak2 over-expressed H9c2 cells was suppressed by TSA or HDAC2 siRNA knockdown (Figure 4C–E). These results suggest that HDAC2 might act as the downstream effector of Jak2 to endorse cardiac hypertrophy response, and Jak2-mediated cardiac hypertrophy is associated with HDAC2 nuclear exportation.

## 3. Discussion

Pressure overload and other stresses promote pathological cardiac hypertrophy, causing heart failure and lethal cardiac arrhythmias. Jak2 plays an important role in the development of cardiac hypertrophy [16,17]. Pressure overload increases the level of circulating Ang-II, which enhances the activity of Jak2 to activate downstream intracellular signaling, eventually promoting expression of fetal type genes (*i.e.*, *ANP* and *BNP*) and accelerating protein synthesis [18].

It has also been suggested that HDAC2 is an important player in cardiac hypertrophy [19,20]. HDAC inhibitors have shown to alleviate the hypertrophic response both *in vivo* and *in vitro* [7,21,22]. In this study, we found that chemical HDAC inhibition by TSA prevented the re-expression of fetal genes and attenuated cardiac hypertrophy in hearts exposed to hypertrophic stimuli (AB). *In vitro*, TSA as well as HDAC2 siRNA knockdown dramatically suppressed Ang-II-induced the re-expression of fetal genes and protein synthesis. These results suggest that HDAC2 is an important molecular target of cardiac hypertrophy. It has been suggested that HDAC2 nuclear export is important for hypertrophic response [23]. Significantly, we found that AB-induced HDAC2 nuclear exportation in hearts, which was almost blocked by Jak2 inhibitor AG-490, the latter also prevented cardiac hypertrophy. *In vitro*, HDAC2 nuclear exportation was also seen in Ang-II-treated H9c2 cells, which was again inhibited by AG-490 or Jak2 genetic silencing. These results suggest that both Jak2 and HDAC2 are important mediators of cardiac hypertrophy response, and Jak2 appears required for HDAC2 nuclear exportation.

It should be noted that Ang-II-induced *ANP*/*BNP* expression in Jak2 over-expressed H9c2 cells was suppressed, but not abolished, by TSA or HDAC2 siRNA knockdown. These results indicate that HDAC2 is an important molecule, but probably not the only one, required in mediating Ang-II-induced fetal gene expression. Other pathways beside HDAC2 could also be involved in Jak2-evoked responses, which require further investigation.

## 4. Materials and Methods

### 4.1. Reagents and Antibodies

TSA, Ang II and AG-490 were purchased from Sigma (Shanghai, China). Antibodies against HDAC2, Lamin-B1, glyceraldehyde-3-phosphate dehydrogenase (GAPDH) and Jak2 were purchased from Cell Signaling Tech (Denver, MA, USA). Antibodies against ANP and BNP were purchased from Santa Cruz Biotech (Santa Cruz, CA, USA).

### 4.2. H9c2 Cardiomyocytes Culture

Rat embryonic ventricular H9c2 cardiomyocytes, purchased from Cell Bank of Shanghai Institute of Biological Science (Shanghai, China), were maintained in Dulbecco’s Modified Eagle’s Medium (DMEM) medium (Invitrogen, Carlsbad, CA, USA), supplemented with a 10% fetal bovine serum (FBS, Invitrogen, Carlsbad, CA, USA) Penicillin/Streptomycin (Sigma, Shanghai, China), and 4 mM l-glutamine (Sigma, Shanghai, China), in a CO_2_ incubator (Sanyo, Shanghai, China) at 37 °C. This cell line has been verified to be the correct lineage and uncontaminated by other cell types or organisms by the supplier.

### 4.3. Mouse Cardiomyocytes Isolation and Culture

Culture of neonatal mouse cardiomyocytes was prepared by methods described previously [24,25]. Briefly, ventricles from C57B6 mice born within 24 h were minced in a nominally Ca^2+^ and Mg^2+^ free Hanks balance solution (Gibco: Biocult Ltd., Paisley, Scotland). Cardiomyocytes were dispersed by the addition of 0.625 mg/mL collagenase (Sigma, Shanghai, China) and incubated at 37 °C for 40 min. Cell suspension was filtered through a polypropylene macroporous filter (mesh opening 105 μm, Shanghai Mai-sheng Biotech, Shanghai, China) and centrifuged at 1200 rpm for 5 min to obtain a cell pellet. Cells were then suspended in M-199 medium supplemented with 5% FBS and 5 mmol/L d-glucose, and pre-plated for 40 min to remove non-cardiomyocytes. The cardiomyocytes were plated at a density of 2 × 10^6^ cells/mL in M-199 supplemented with 10% FBS on 18-mm square cover-slips or in 35-mm petri dishes pre-coated with 1% gelatin (Sigma, Shanghai, China). Cells were incubated at 37 °C in a humidified atmosphere containing 5% CO_2_. A confluent monolayer of spontaneously beating cells was formed within 2 days.

### 4.4. DNA Constructs and Transfection

PCR amplified rat Jak2 or HDAC2 cDNA (purchased from and verified by Shanghai Kaiji Biotech, Shanghai, China) were cloned into pcDNA3.1-GFP (Invitrogen, Carlsbad, CA, USA). The empty vector or cDNA plasmid (0.5 μg/mL) was transfected into cultured H9c2 cardiomyocytes cells through Lipofectamine 2000 (Invitrogen, Carlsbad, CA, USA) according to manufacturer’s protocol. The stable clones expressing targeted cDNA or the empty vector were selected by G-418 (200 μg/mL, Sigma, Shanghai, China) for 2–3 weeks. Expressions of the targeted protein as well as equal loading (GAPDH) in the stable cells were tested by Western blots.

### 4.5. Quantitative Real-Time PCR

Total RNA was prepared using TRIzol reagent (Invitrogen, Carlsbad, CA, USA), and the reverse transcription polymerase chain reaction (RT-PCR) was performed using TOYOBO ReverTra Ace-a RT-PCR kit (Toyobo, Osaka, Japan) according to the manufacturer’s instructions. The real-time PCR was performed on a Bio-Rad IQ5 multicolor detection system (Bio-Rad, Hercules, CA, USA) by using synthesized cDNA. All real-time PCRs were performed at least triplicate. Primers were follows: For *ANP* gene, sense, 5'-GGTGTCCAACACAGATCTGA-3' and antisense, 5'-CCACTAGACCACTCATCTAC-3'; For *BNP* gene, sense, 5'-TATAAAAGGCAGAGGCACCG-3' and antisense, 5'-AATCATCTGGGACAGCACCT-3'; For *GAPDH* gene, sense, 5'-ACCACAGTCCATGCCATCAC-3' and antisense, 5'-TCCACCACCCTGTTGCTGTA-3'; For *HDAC2* gene, sense, 5'-GCTATTCCAGAAGATGCTGTTC-3', antisense, 5'-GTTGCTGAGCTGTTCTGATTTG-3'; For *Jak2* gene, sense, 5'-GCTACATCCATCTACCTCAGTTTCC-3', antisense, 5'-ATTCCAATGTTATGTTGAACCTGCC-3'. One RNA sample of each preparation was processed without real time-reaction to provide a negative control in subsequent PCR. After amplification, melt curve analysis was performed to analyze product melting temperature. *GAPDH* gene was chosen as the reference gene for normalization, and the 2^−∆∆*C*t (Cycle Threshold)^ method [26] was applied to quantify targeted mRNA change within samples. The fold change of targeted mRNA expression = 2^−∆∆*C*t^. Where ∆∆*C*_t_ = (*C*_t_ targeted gene − *C*_t_ GAPDH) of treatment group − (*C*_t_ targeted gene − *C*_t_ GAPDH) of control group.

### 4.6. Western Blots

After treatment, cells and homogenized tissues were incubated in RIPA buffer containing 50 mM *N*-2-hydroxyethylpiperazine-*N*-2'-ethanesulfonic acid (HEPES, pH 7.0), 250 mM NaCl, 0.1% Nonidet P-40 and 1 mM phenylmethylsulfonylfluoride (All from Sigma, Shanghai, China), followed by incubation on ice for 10 min. Subsequently, lysates were sonicated for 10 s. The extracts were cleaned by centrifugation at 15,000 rpm for 10 min at 4 °C and the supernatants were harvested. The protein concentration was measured. Thirty-μg protein samples were size-fractionated to a single dimension by sodium dodecyl sulfate-polyacrylamide gel electrophoresis (SDS-PAGE) (10% gels) and transferred onto a 0.45-μm polyvinylidene difluoride (PVDF) membrane (Millipore, Billerica, MA, USA). The blot was then washed 3 times in Tris-buffered saline with 0.1% Tween-20 (TBST, Sigma, Shanghai, China) and incubated for 1 h at room temperature in blocking buffer (10% milk (Sigma, Shanghai, China) in TBST). Subsequently, the blot was immuno-blotted with an appropriate primary antibody overnight at 4 °C, followed by a horseradish peroxidase (HRP)-conjugated secondary antibody incubation for 1 h at room temperature. Reaction products were detected by the enhanced chemiluminescence system (ECL, Amersham, Buckinghamshire, UK). The intensity of each indicated band was normalized with the loading control. The value was expressed as fold change *vs*. the band labeled as “1.00”. Nuclear fractions were extracted through the nuclear extraction kit from Merck (Shanghai, China) according to the instruction manual.

### 4.7. RNA Interference (RNAi)

SiRNA (100 nM) was transfected to cultured H9c2 cells (50% confluence in OptiMEM) through Lipofectamine RNAi-MAX Transfection Reagent (Invitrogen, Carlsbad, CA, USA) according to the manufacturer’s protocol, control cells were transfected with same concentration (100 nM) of scramble siRNA (Santa Cruz Biotech, Santa Cruz, CA, USA). After transfection, cells were further cultured for 48 h. Expression of targeted protein in transfected cells was always tested by Western blots. The siRNA sequences specific to rat Jak2 or HDAC2 were designed and verified by Dharmacon (siGenome™, Dharmacon, Lafayette, IN, USA).

### 4.8. Aortic Banding (AB) Operation in Mice

Male C57B6 mice, aged 8–10 weeks, were subjected to the AB operation for two weeks to produce pressure overload [27]. Briefly, after anesthetization (ketamine, 25 mg/kg/IP, Sigma, Shanghai, China) and artificial ventilation, the transverse aorta was banded with a 7-0 nylon suture by binding the aorta together with a blunted 26-gauge needle, which was pulled out later. AG-490 (1 mg/kg body weight per day), TSA (0.6 mg/kg/day) or PBS (vehicle) was continuously administered by Alzet osmotic mini-pumps (Model 2002, DURECT, Cupertino, CA, USA) implanted subcutaneously into the back of mice from 3 days before AB until AB was finished. Animals recovered for 2 weeks after AB to develop cardiac hypertrophy. All animals were maintained in accordance with the guidelines of the National Institute of Health (NIH) (Guide for the Care and Use of Laboratory Animals, 1996), the European Communities Council. The protocol is approved by Animal Care and Use Committee of all authors’ institutions (approved at 10 November 2011, identification code: SHXQYY-100015, contact name of institutional review board: Shen Wang).

### 4.9. Transthoracic Echocardiogram

Transthoracic Echocardiogram (TTE) was performed as previously described by using an animal specific instrument (VisualSonics Inc., Toronto, ON, Canada) [28,29]. Mice were anesthetized and M-mode images of short axis view of the LV were recorded when the mice partially recovered from anesthesia. All measurements were averaged for five consecutive cardiac cycles and were carried out by three experienced independent technicians.

### 4.10. Morphological Analyses

Mice were sacrificed and hearts were excised two weeks after AB. The hearts were perfused with PBS followed by 4% poly-formaldehyde (Sigma, Shanghai, China). For histological analysis, heart tissues were fixed in 10% formalin (Sigma, Shanghai, China) and embedded in paraffin (Sigma, Shanghai, China), sectioned at 4 μm thickness and stained with hematoxylin and eosin (H&E) (Sigma, Shanghai, China). Digital photographs were taken at magnification of 400×. For measurement, five random high-power fields from each section were chosen and quantified. The cross-sectional area (CSA) of cardiomyocytes was analyzed and quantitatively by morphology meter of H&E stained sections through an automated image analysis system (Image-Pro Plus 5.0, Media, Cybernetics, Sliver Spring, MD, USA).

### 4.11. [^3^H] Leucine Incorporation

Cultured neonatal mouse cardiomyocytes were incubated with [^3^H] leucine (1 μCi/mL) DMEM. (Invitrogen, Carlsbad, CA, USA) Cardiomyocytes were treated with 5% Trichloroacetic acid (Sigma, Shanghai, China). The protein precipitates were dissolved in 1 mL of 100 mM/L sodium hydroxide, and radio activities were determined with a liquid scintillation counter.

### 4.12. Statistical Analysis

Data were expressed as means ± SEM, multiple group comparison was performed by the Bonferroni method (SPSS 13.0, Chicago, IL, USA). Comparison between two groups under identical conditions was performed by the two-tailed Student’s *t*-test (Excel 2007). A value of *p* < 0.05 was considered statistically significant.

## 5. Conclusions

In summary, these results suggest that pressure overload-induced cardiac hypertrophy response requires Jak2-HDAC2 signaling.
